# Epidemiological characteristics of seven notifiable respiratory infectious diseases in the mainland of China: an analysis of national surveillance data from 2017 to 2021

**DOI:** 10.1186/s40249-023-01147-3

**Published:** 2023-11-13

**Authors:** Le-le Deng, Ya-jun Han, Zhuo-wei Li, Da-yan Wang, Tao Chen, Xiang Ren, Guang-xue He

**Affiliations:** 1 National Institute for Viral Disease Control and Prevention, Chinese Center for Disease Control and Prevention, Beijing, 102206 China; 2 Division of Infectious Disease, Key Laboratory of Surveillance and Early-Warning On Infectious Disease, Chinese Center for Disease Control and Prevention, Beijing, 102206 China

**Keywords:** Respiratory infectious diseases, Surveillance, Epidemiological characteristics, Incidence, China

## Abstract

**Background:**

Respiratory infectious diseases (RIDs) remain a pressing public health concern, posing a significant threat to the well-being and lives of individuals. This study delves into the incidence of seven primary RIDs during the period 2017–2021, aiming to gain deeper insights into their epidemiological characteristics for the purpose of enhancing control and prevention strategies.

**Methods:**

Data pertaining to seven notifiable RIDs, namely, seasonal influenza, pulmonary tuberculosis (PTB), mumps, scarlet fever, pertussis, rubella and measles, in the mainland of China between 2017 and 2021 were obtained from the National Notifiable Disease Reporting System (NNDRS). Joinpoint regression software was utilized to analyze temporal trends, while SaTScan software with a Poisson probability model was used to assess seasonal and spatial patterns.

**Results:**

A total of 11,963,886 cases of the seven RIDs were reported during 2017–2021, and yielding a five-year average incidence rate of 170.73 per 100,000 individuals. Among these RIDs, seasonal influenza exhibited the highest average incidence rate (94.14 per 100,000), followed by PTB (55.52 per 100,000), mumps (15.16 per 100,000), scarlet fever (4.02 per 100,000), pertussis (1.10 per 100,000), rubella (0.59 per 100,000), and measles (0.21 per 100,000). Males experienced higher incidence rates across all seven RIDs. PTB incidence was notably elevated among farmers and individuals aged over 65, whereas the other RIDs primarily affected children and students under 15 years of age. The incidences of PTB and measles exhibited a declining trend from 2017 to 2021 (APC = −7.53%, *P* = 0.009; APC = −40.87%, *P* = 0.02), while the other five RIDs peaked in 2019. Concerning seasonal and spatial distribution, the seven RIDs displayed distinct characteristics, with variations observed for the same RIDs across different regions. The proportion of laboratory-confirmed cases fluctuated among the seven RIDs from 2017 to 2021, with measles and rubella exhibiting higher proportions and mumps and scarlet fever showing lower proportions.

**Conclusions:**

The incidence of PTB and measles demonstrated a decrease in the mainland of China between 2017 and 2021, while the remaining five RIDs reached a peak in 2019. Overall, RIDs continue to pose a significant public health challenge. Urgent action is required to bolster capacity-building efforts and enhance control and prevention strategies for RIDs, taking into account regional disparities and epidemiological nuances. With the rapid advancement of high-tech solutions, the development and effective implementation of a digital/intelligent RIDs control and prevention system are imperative to facilitate precise surveillance, early warnings, and swift responses.

**Graphical Abstract:**

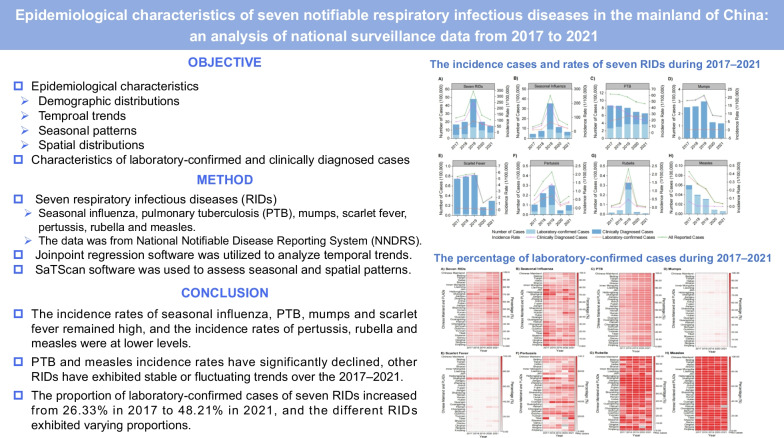

**Supplementary Information:**

The online version contains supplementary material available at 10.1186/s40249-023-01147-3.

## Background

Given the distinct transmission routes and modes of infection associated with respiratory infectious diseases (RIDs), many emerging and re-emerging RIDs have the potential to spread rapidly among susceptible populations worldwide. These RIDs persist as a significant public health challenge, imposing a global disease burden and jeopardizing individuals’ health and well-being [1, 2]. In the mainland of China, thirteen RIDs are subject to statutory reporting, with seasonal influenza and pulmonary tuberculosis (PTB) emerging as the most prevalent RIDs in recent decades [3]. In 2020, reported influenza morbidity stood at 81.58 per 100,000 individuals in the mainland of China, with a reported mortality rate of 0.005 per 100,000 [4]. Concurrently, PTB's notifiable incidence rate was 47.76 per 100,000, accompanied by a reported mortality rate of 0.14 per 100,000 [4]. Mumps, scarlet fever, pertussis, rubella and measles followed suit, representing five RIDs with comparatively elevated annual incidence rates that often clustered within specific regions [5–12]. Furthermore, the emergence and re-emergence of RIDs in recent years underscore the need for strengthen surveillance to enable early warnings and swift, effective responses. Consequently, a comprehensive elucidation of the demographic, temporal, seasonal, and spatial distribution characteristics of RIDs is crucial for the development of targeted, efficient interventions to curtail their propagation.

While several studies have explored the epidemiological features of various RIDs across different time frames and geographical locations in China [6–14], the present study stands apart due to its unique data sources and temporal scope. Leveraging data from the National Notifiable Disease Reporting System (NNDRS), this study provides a more comprehensive analysis, incorporating recent data and encompassing factors such as case categories (clinically diagnosed and laboratory-confirmed), gender, and occupation. The study’s focus centers on the demographic, temporal, seasonal, and spatial distribution characteristics of seven RIDs during the period 2017–2021, with the aim of furnishing valuable insights to inform effective control and prevention strategies.

## Methods

### Data collection

This study draws upon surveillance data for seven RIDs in the mainland of China spanning the years 2017 to 2021, sourced from the NNDRS. Among these RIDs, PTB, pertussis, measles, and scarlet fever are classified as category B infectious diseases, while seasonal influenza, mumps, and rubella fall under category C infectious diseases. The NNDRS is an internet-based real-time disease-reporting system that encompasses various healthcare facilities (community health centers, township health centers, and village clinics) across the mainland of China, boasting coverage of 55,077 health facilities in 31 provincial-level administrative divisions (PLADs) [15, 16]. The anonymized data for each reported case were compiled, encompassing demographic details (residential ID number, sex, age, and occupation) and clinical particulars (dates of symptom onset, diagnosis date, diagnosis category). Reported cases encompass both clinically diagnosed cases and laboratory-confirmed cases, aligned with the diagnosis criteria stipulated and disseminated by the National Health Commission of the People’s Republic of China [15, 16]. Clinically diagnosed cases are established based on primary symptoms, signs, and epidemiological links. In contrast, laboratory-confirmed cases entail a synthesis of clinical diagnosis and corroborating laboratory testing [15, 16].

Demographic data by age and sex for 31 PLADs and the country were culled from the National Bureau of Statistics of China (http://www.stats.gov.cn/english/Statisticaldata/AnnualData, accessed on April 20, 2023) [17]. The standard base map of China [GS(2019)1822] was sourced from the Standard Map Service (http://bzdt.ch.mnr.gov.cn/, accessed on April 20, 2023) under the Ministry of Natural Resources of the People's Republic of China.

### Descriptive analysis

Incidence rates for both national and provincial levels were calculated on a monthly and annual basis, along with stratifications by sex and age groups. Visual representations in the form of bar graphs and scatter line plots were employed to depict trends in cases and incidence rates. Stacked plots and heat maps elucidated trends in occupation proportions and laboratory-confirmed cases, respectively. Seasonal attributes were visualized via radar charts, generated using SaTScan (version 10.1, Kulldorff and Information Management Services, Inc., Boston, MA, USA) outputs. Figures were crafted using R software with the ggplot2 package (version 4.0.0, R Development Core Team 2020) and OriginPro (version 2021, OriginLab Corporation, Northampton, MA, USA), while spatial characteristics were mapped using ArcGIS software (version 10.7, ESRI, Redlands, CA, USA).

### Joinpoint regression analysis

Temporal trends were subjected to analysis using Joinpoint regression software (version 4.9.0.0, National Cancer Institute, Rockville, MD, USA) [18]. The default modeling method was the grid search method (GSM), while the Monte Carlo permutation test served as the default optimization strategy for the model. The Bayesian information criterion (BIC) was employed as a metric for gauging good fit [18]. The annual percent change (APC) serves as an indicator of the average annual percentage alteration in incidence rates and is represented by the slope of the fitted line. An APC > 0 (*P* < 0.05) denotes an increasing trend in incidence rates, whereas an APC < 0 (*P* < 0.05) signifies a decreasing trend. Conversely, trends lacking significant changes are denoted by APC values falling outside these ranges [13]. The APC is calculated using the following formula:

$$\ln (y) = \beta_{0} + \beta_{1} x$$

$$APC = \left[ {\frac{{{\text{y}}_{x + 1} - y_{x} }}{{y_{x} }}} \right] \times 100 = (e^{{\beta_{1} }} - 1) \times 100$$

Note:* y* is incidence rate, *x* is year, *β*_1_ is regression coefficient.

### Seasonal and spatial analysis

The examination of seasonal and spatial characteristics was conducted using the SaTScan software. This software employed a Poisson probability model to identify clusters of RIDs in terms of seasonality, with a temporal window encompassing 50%. The software detected spatial clusters of RIDs in different regions for the years 2017 and 2021, employing the Poisson probability model and a spatial window covering 50% of the study areas [19]. In order to avoid overlap of PLADs in clusters, we adjust the default parameter to remove “Gini Optimized Cluster Collection” and set “No Geographical Overlap” in clusters [19]. By juxtaposing observed and predicted events within each location window, assuming a random distribution, probable clusters were pinpointed. The cluster exhibiting the highest log-likelihood ratio (*LLR*) was deemed the most likely cluster, while others were ranked as secondary clusters in a specific sequence [19]. The concept of relative risk (*RR*) denoted the ratio of estimated risks within and outside the cluster, serving as an indicator of the elevated infection risk faced by individuals residing within the cluster compared to those outside it [19].

In the context of the seasonal analysis, the 31 PLADs in the mainland of China were stratified into two distinct regions: southern China (encompassing Anhui, Fujian, Guangdong, Guangxi, Guizhou, Hainan, Hubei, Hunan, Jiangsu, Jiangxi, Shanghai, Sichuan, Yunnan, Zhejiang and Chongqing) and northern China (covering Beijing, Gansu, Hebei, Henan, Heilongjiang, Jilin, Liaoning, Inner Mongolia, Ningxia, Qinghai, Shandong, Shanxi, Shaanxi, Tianjin, Tibet and Xinjiang). This classification was based on meteorological attributes [16].

## Results

### Overview of RIDs

**Fig. 1:**
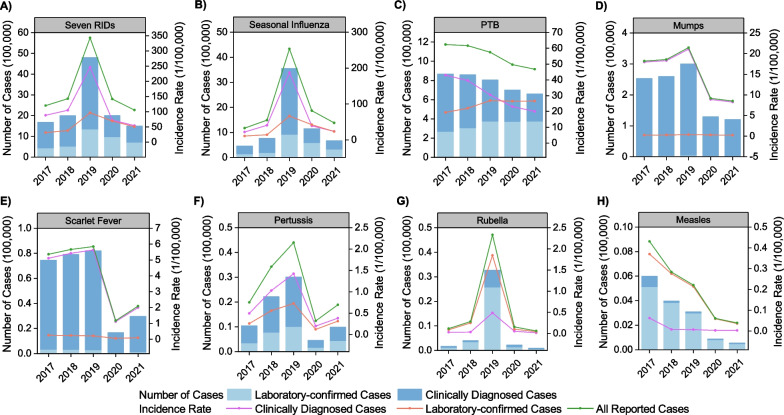
The incidence cases and rates of seven RIDs during 2017–2021. Notes: Seven RIDs includes seasonal influenza, PTB, mumps, scarlet fever, pertussis, rubella and measles

A total of 11,963,886 cases of seven RIDs were reported in the mainland of China between 2017 and 2021. Of these, 7,941,892 (66.38%) were clinically diagnosed cases, while 4,021,994 (33.62%) were laboratory-confirmed cases. The five-year average incidence rate for all seven RIDs combined was 170.73 cases per 100,000 population. The highest incidence rate was recorded in 2019, reaching 343.18 cases per 100,000 (Fig. 1). Examining the individual RIDs, the highest five-year average incidence rate was observed for seasonal influenza (94.14 per 100,000), followed by PTB (55.52 per 100,000), mumps (15.16 per 100,000), scarlet fever (4.02 per 100,000), pertussis (1.10 per 100,000), rubella (0.59 per 100,000), and measles (0.21 per 100,000) (Fig. 1).

### Population distributions of RIDs

**Fig. 2:**
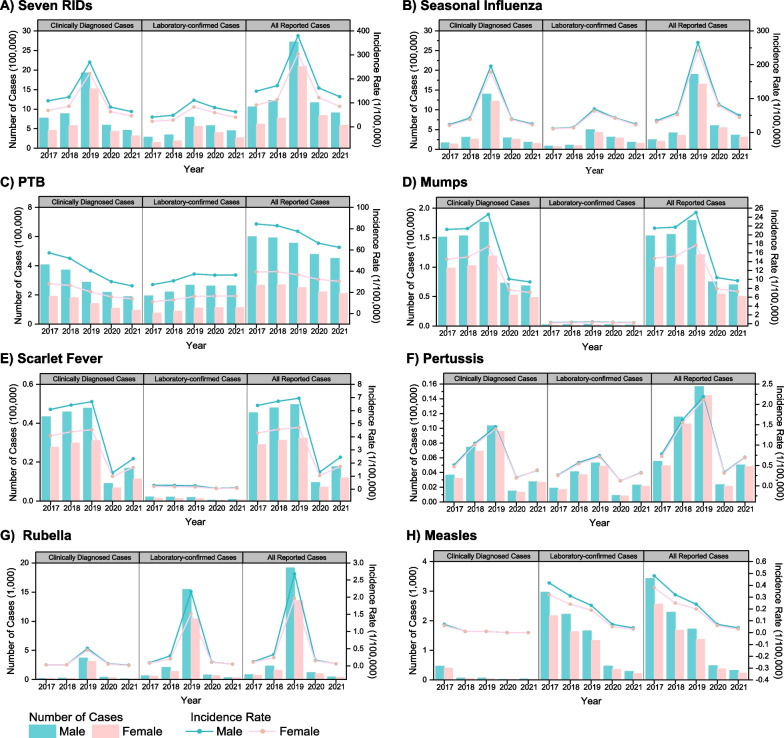
The incidence cases and rates of seven RIDs by gender during 2017–2021

**Fig. 3:**
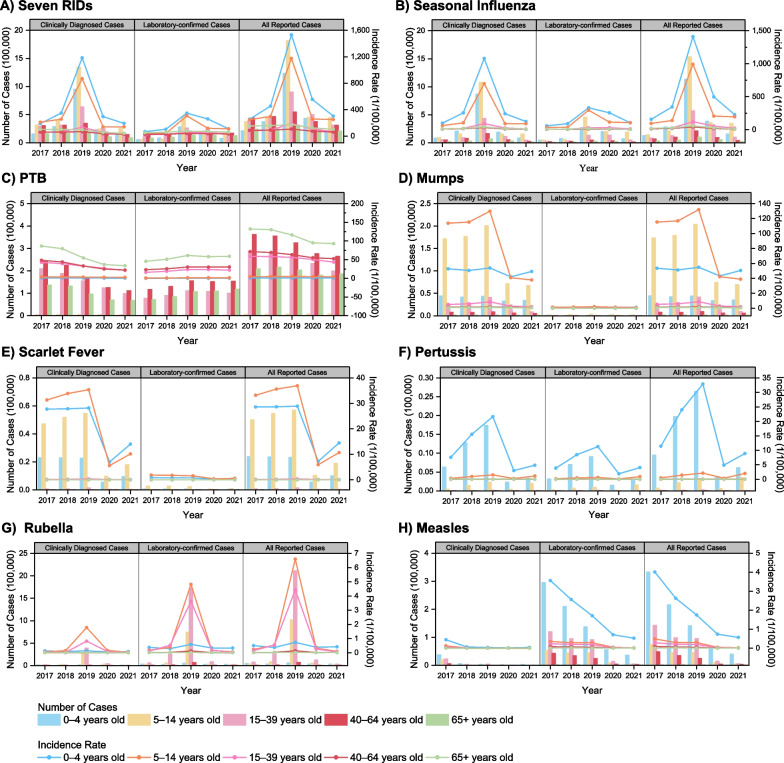
The incidence cases and rates of seven RIDs by age groups during 2017–2021

Across all seven RIDs, males exhibited higher incidence cases and rates compared to females, particularly notable for PTB, mumps, and scarlet fever, as evident in both clinically diagnosed and laboratory-confirmed cases (Fig. 2). Age distribution revealed that the 0–4 and 5–14 age groups experienced the highest incidence rates, a pattern consistent across laboratory-confirmed and clinically diagnosed cases (Fig. 3A). Notably, seasonal influenza predominantly affected the 5–14 age group, with the highest incidence rate occurring in the 0–4 age group (Fig. 3B). For PTB, the highest incidence rate was observed among those aged 65 years and older (Fig. 3C). The 40–64 age group accounted for the highest number of PTB cases (39.62–41.82% during 2017–2021), followed by the 15–39 age group (30.20–33.31%). The 5–14 age group showed the highest incidence rates and cases for mumps and scarlet fever (Fig. 3D, E). Pertussis primarily affected children in the 0–4 age group, with the highest incidence rates and cases in that group (Fig. 3F). Rubella cases were concentrated in the 15–39 age group, with the highest incidence rate observed in the 5–14 age group (Fig. 3G). Measles cases were most frequent in the 0–4 age group, followed by the 15–39 age group (Fig. 3H).

### Occupational distribution of populations

**Fig. 4:**
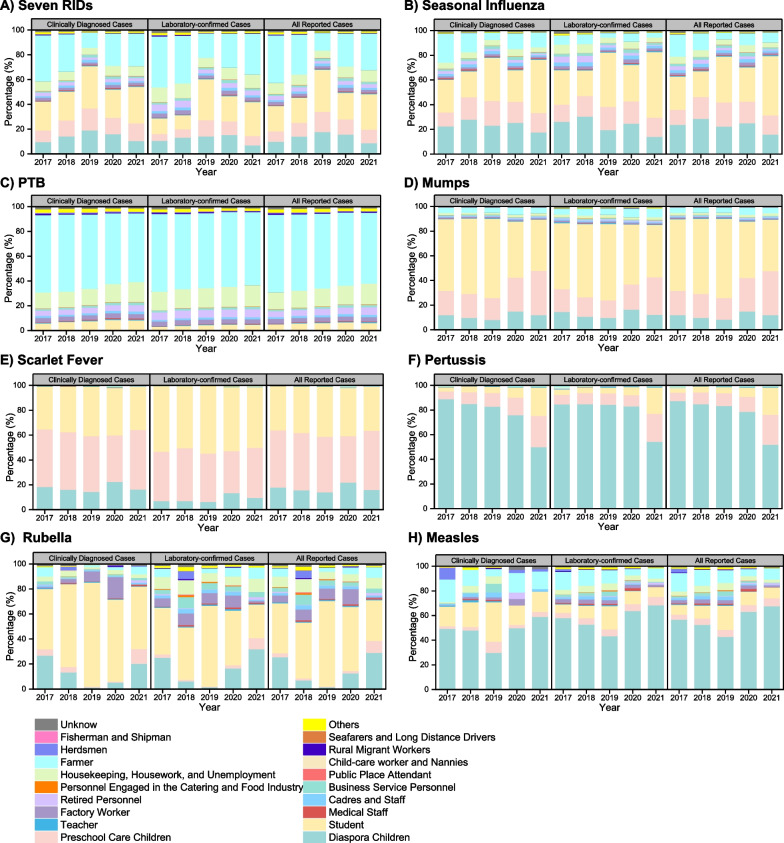
The percentage of occupations for seven RIDs during 2017–2021. *RIDs*: Respiratory infectious diseases; *PTB*: Pulmonary tuberculosis

Analyzing twenty occupational categories based on NNDRS classification (Fig. 4), students, farmers, diaspora children and preschool care children emerged as the predominant populations, accounting for 26.90%, 24.58%, 14.71% and 13.07% respectively, across all seven RIDs (Fig. 4A). This trend remained consistent for specific RIDs, with students comprising the dominant group for seasonal influenza, mumps, scarlet fever and rubella (Fig. 4B, D, E, G), while diaspora children took the lead for pertussis and measles (Fig. 4F, H). Notably, farmers constituted the primary population for PTB, trailed by housekeepers, house-workers and the unemployed (Fig. 4C).

### Temporal trends of RIDs

**Fig. 5:**
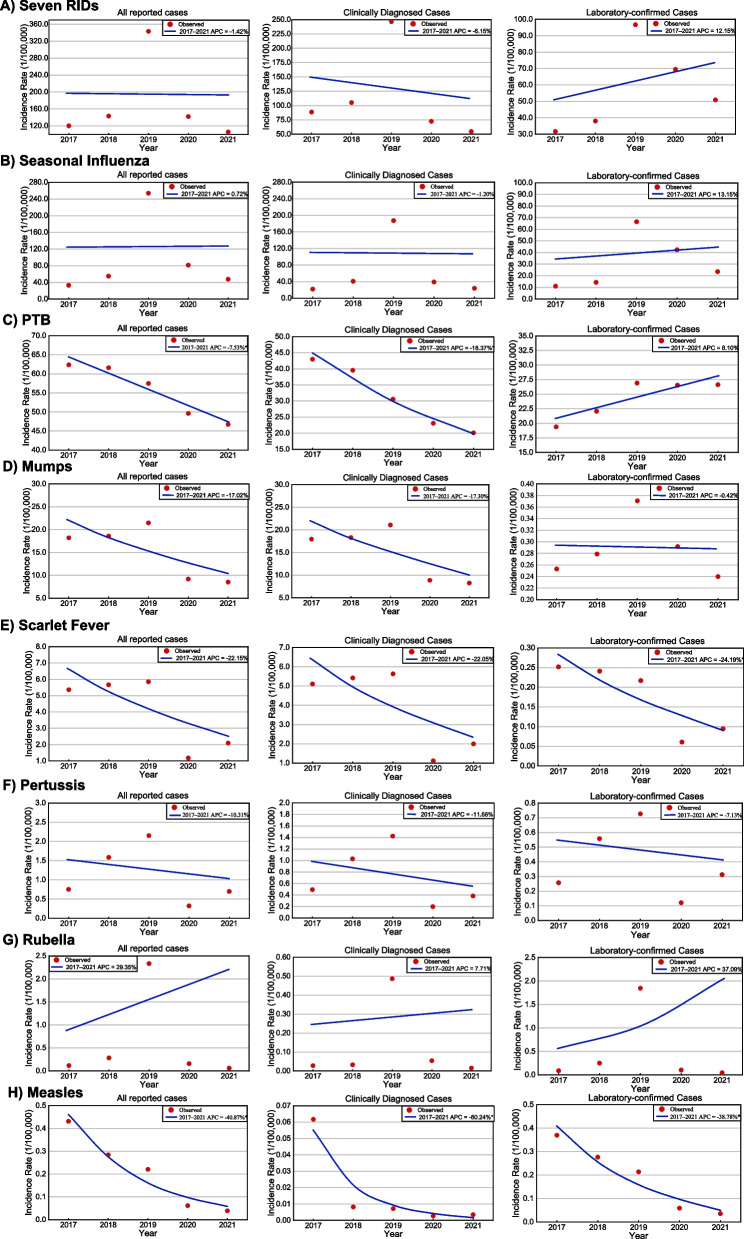
The temporal trends of incidence rates for seven RIDs during 2017–2021. Notes: Point represent the observed incidence rates, line represent the fitting line of the observed incidence rates and the slope indicating the value of APC; * represent the *P* < 0.05. *RIDs*: Respiratory infectious diseases; *PTB*: Pulmonary tuberculosis

The incidence rates of the seven RIDs exhibited a peak in 2019, although no significant temporal trend was observed (APC = −1.42%, 95% *CI*: −39.14 to 57.09%, *P* = 0.96) (Table 1 and Fig. 5A). The incidence of laboratory-confirmed RIDs cases increased (APC = 12.15%, 95% *CI*: −18.67–59.49%, *P* = 0.56), while clinically diagnosed cases decreased (APC = −6.15%, 95% *CI*: −45.43 to 53.48%, *P* = 0.84), though neither trend proved statistically significant (Table 1 and Fig. 5A). Seasonal influenza, mumps, scarlet fever, pertussis and rubella all displayed peak incidences in 2019 without significant temporal trends (Table 1 and Fig. 5B, D–G). However, PTB and measles demonstrated decreasing incidence rates from 2017 to 2021. The PTB incidence rate notably decreased from 62.36/100,000 in 2017 to 46.72/100,000 in 2021, reflecting an APC of −7.53% (95% *CI*: −12.31 to −2.79%, *P* = 0.009), with clinically diagnosed rates also decreasing significantly (APC = −18.37%, 95% *CI*: −26.83 to −10.76%, *P* = 0.002) (Table 1 and Fig. 5C). The measles incidence rate underwent a substantial decline from 0.43/100,000 in 2017 to 0.04/100,000 in 2021, with an APC of −40.87% (95% *CI*: −61.18 to −24.32%, *P* = 0.02), and similar patterns emerged for laboratory-confirmed rates (APC = −38.78%, 95% *CI*: −58.00 to −22.20%, *P* = 0.03) and clinically diagnosed rates (APC = −60.24%, 95% *CI*: −86.61 to −44.67%, *P* = 0.02) (Table 1 and Fig. 5H).

**Table 1:** The APC and *CI* for incidence of seven RIDs in the mainland of China during 2017–2021

RIDs	All reported cases			Clinically diagnosed cases			Laboratory-confirmed cases		
	Joinpoint Year Range	APC (95% *CI*)	*P*	Joinpoint Year Range	APC (95% *CI*)	*P*	Joinpoint Year Range	APC (95% *CI*)	*P*
Seven RIDs	2017–2021	−1.42 (−39.14 to 57.09)	0.96	2017–2021	−6.15 (−45.43 to 53.48)	0.84	2017–2021	12.15 (−18.67 to 59.49)	0.56
Seasonal Influenza	2017–2021	−0.72 (−54.07 to 130.48)	0.99	2017–2021	−1.20 (−61.99 to 152.95)	0.98	2017–2021	13.15 (−26.98 to 90.42)	0.72
PTB	2017–2021	−7.53 (−12.31 to −2.79)	0.009	2017–2021	−18.37 (−26.83 to −10.76)	0.002	2017–2021	8.10 (−0.46 to 17.94)	0.06
Mumps	2017–2021	−17.02 (−36.50 to 3.32)	0.19	2017–2021	−17.30 (−37.36 to 3.71)	0.19	2017–2021	−0.42 (−17.65 to 19.85)	0.96
Scarlet fever	2017–2021	−22.15 (−53.11 to 11.09)	0.21	2017–2021	−22.05 (−53.59 to 12.28)	0.22	2017–2021	−24.19 (−45.40 to −5.15)	0.02
Pertussis	2017–2021	−10.31 (−66.13 to 90.68)	0.72	2017–2021	−11.68 (−63.15 to 77.21)	0.70	2017–2021	−7.13 (−60.92 to 83.31)	0.78
Rubella	2017–2021	29.35 (−74.18 to 540.89)	0.81	2017–2021	7.71 (−75.11 to 362.20)	0.94	2017–2021	37.09 (−76.49 to 787.58)	0.77
Measles	2017–2021	−40.87 (−61.18 to −24.32)	0.02	2017–2021	−60.24 (−86.61 to −44.67)	0.02	2017–2021	−38.78 (−58.00 to −22.20)	0.03

APC annual percentage changes; *CI* confidence interval; RIDs respiratory infectious diseases; PTB pulmonary tuberculosis. Notes: The unit of APC is %

### Seasonal patterns of RIDs

**Fig. 6:**
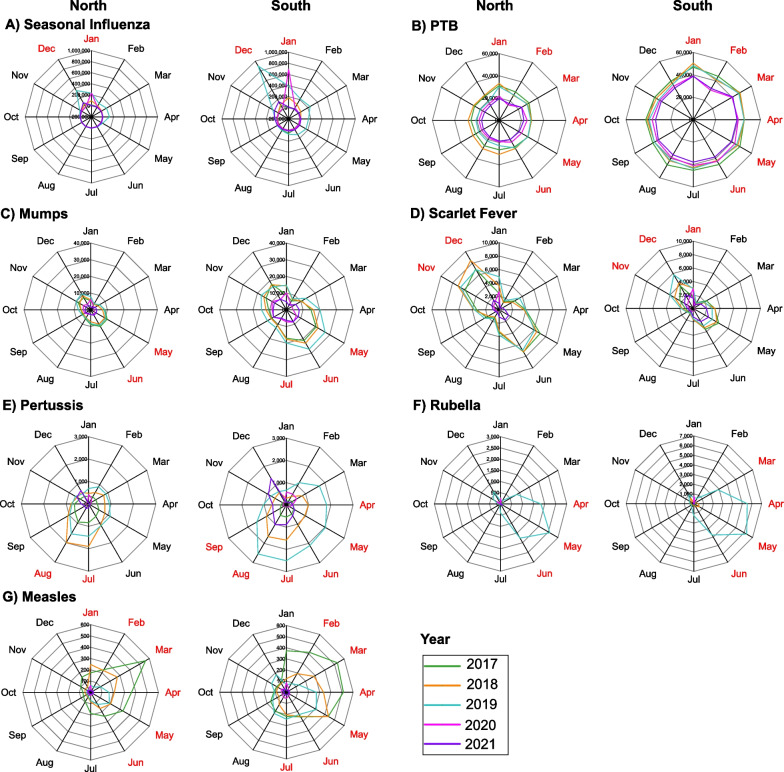
The seasonal distributions of incidence rates for seven RIDs during 2017–2021. Notes: Red represent months with high incidence rate. *RIDs*: Respiratory infectious diseases; *PTB*: Pulmonary tuberculosis

Seasonal characteristics of the seven RIDs exhibited variations, including divergent patterns within the same RIDs across different areas (Fig. 6). Seasonal influenza incidence rates peaked in December and January for both northern and southern China (Fig. 6A). PTB displayed higher rates between January and June in both regions (Fig. 6B). Mumps, scarlet fever, pertussis, and rubella had extended high-incidence periods in southern China compared to the north (Fig. 6C–F). Measles incidence was prominent from January to June in the north, and from February to July in the south (Fig. 6G).

### Spatial distributions of seven RIDs

The geographic distribution of RIDs incidence varied (Table 2 and Additional file 1: Fig. S1). In 2017, four clusters covered 10 PLADs for all seven RIDs, with the most likely cluster located mainly in Hainan, Guangxi, Guangdong, Guizhou and Hunan (*RR* = 1.72, *P* < 0.001) (Table 2). In 2021, three clusters spanning 12 PLADs were identified, with the most likely cluster encompassing 10 PLADs (Guizhou, Chongqing, Yunnan, Guangxi, Sichuan, Hunan, Guangdong, Hainan, Hubei and Shaanxi; *RR* = 1.95, *P* < 0.001) (Table 2). Examining trends in all seven RIDs across 31 PLADs from 2017 to 2021, no significant trends were observed (Table 3).

**Table 2:** The spatial and clustered characteristics of seven RIDs in 2017 and 2021

RIDs	2017					2021				
	Clusters	Covered PLADs	*RR*	*LLR*	*P*	Clusters	Covered PLADs	*RR*	*LLR*	*P*
Seven RIDs	Primary cluster	Hainan, Guangxi, Guangdong, Guizhou, Hunan	1.72	47,487.44	< 0.001	Primary cluster	Guizhou, Chongqing, Yunnan, Guangxi, Sichuan, Hunan, Guangdong, Hainan, Hubei, Shaanxi	1.95	82,350.33	< 0.001
	Secondary cluster	Xinjiang, Qinghai, Tibet	2.04	16,058.77	< 0.001	Secondary cluster	Zhejiang	1.27	2183.25	< 0.001
	Tertiary cluster	Beijing	2.01	9837.21	< 0.001	Tertiary cluster	Henan	1.03	37.40	< 0.001
	Fourth cluster	Shaanxi	1.03	15.24	< 0.001					
Seasonal Influenza	Primary cluster	Guangdong	3.68	55,855.78	< 0.001	Primary cluster	Guizhou, Chongqing, Yunnan, Guangxi, Sichuan, Hunan, Guangdong, Hainan, Hubei, Shaanxi	2.42	64,073.84	< 0.001
	Secondary cluster	Beijing	5.73	33,864.64	< 0.001	Secondary cluster	Zhejiang	1.88	8575.22	< 0.001
	Tertiary cluster	Hubei	1.91	5756.80	< 0.001	Tertiary cluster	Henan	1.35	2327.89	< 0.001
	Fourth cluster	Zhejiang	1.68	3342.46	< 0.001	Fourth cluster	Beijing	1.33	505.20	< 0.001
PTB	Primary cluster	Xinjiang, Qinghai, Tibet	3.18	28,897.81	< 0.001	Primary cluster	Guangxi, Hainan, Guizhou, Guangdong, Yunnan, Hunan, Chongqing, Sichuan, Jiangxi, Hubei	1.66	21,239.65	< 0.001
	Secondary cluster	Guangxi, Hainan, Guizhou, Guangdong, Yunnan, Hunan, Chongqing, Sichuan, Jiangxi, Hubei	1.48	16,521.19	< 0.001	Secondary cluster	Xinjiang, Qinghai, Tibet	2.07	6807.50	< 0.001
	Tertiary cluster	Heilongjiang	1.22	505.52	< 0.001	Tertiary cluster	Heilongjiang	1.17	196.48	< 0.001
						Fourth cluster	Liaoning	1.04	15.70	< 0.001
Mumps	Primary cluster	Tibet, Sichuan, Yunnan, Qinghai, Gansu, Chongqing, Guizhou, Xinjiang, Ningxia, Shaanxi, Guangxi, Hunan, Shanxi, Henan, Hainan, Hubei	2.24	19,901.49	< 0.001	Primary cluster	Yunnan, Guizhou, Guangxi, Chongqing, Sichuan, Hainan, Hunan, Guangdong, Shaanxi, Gansu, Tibet, Qinghai, Hubei	1.97	6787.21	< 0.001
						Secondary cluster	Anhui	1.17	67.07	< 0.001
Scarlet Fever	Primary cluster	Heilongjiang, Jilin, Liaoning, Beijing, Tianjin, Shandong, Hebei, Inner Mongolia, Shanxi	2.52	7610.13	< 0.001	Primary cluster	Ningxia, Gansu, Qinghai, Shaanxi, Inner Mongolia, Shanxi	2.13	1077.90	< 0.001
	Secondary cluster	Shanghai	3.45	2184.40	< 0.001	Secondary cluster	Guangxi, Hainan, Guizhou, Guangdong, Yunnan, Hunan	1.45	422.84	< 0.001
	Tertiary cluster	Gansu, Qinghai, Ningxia, Shaanxi	1.45	336.60	< 0.001	Tertiary cluster	Shandong, Tianjin	1.47	204.23	< 0.001
	Fourth cluster	Guangdong	1.12	36.08	< 0.001	Fourth cluster	Shanghai	1.91	162.21	< 0.001
						Fifth cluster	Jiangsu	1.13	13.03	< 0.001
Pertussis	Primary cluster	Shandong, Tianjin	8.04	4364.84	< 0.001	Primary cluster	Chongqing, Sichuan, Guizhou, Shaanxi, Yunnan, Hunan	3.66	1944.63	< 0.001
	Secondary cluster	Chongqing	4.93	801.93	< 0.001	Secondary cluster	Shaanxi	2.13	109.29	< 0.001
	Tertiary cluster	Guangdong	1.37	44.38	< 0.001					
Rubella	Primary cluster	Fujian	4.53	128.27	< 0.001	Primary cluster	Qinghai	42.06	343.41	< 0.001
	Secondary cluster	Xinjiang, Qinghai	2.85	36.95	< 0.001	Secondary cluster	Guizhou, Chongqing, Yunnan	2.1	26.54	< 0.001
	Tertiary cluster	Hebei, Shanxi, Tianjin, Beijing	1.79	34.49	< 0.001	Tertiary cluster	Inner Mongolia	2.41	9.81	< 0.001
						Fourth cluster	Hunan	1.69	7.07	
Measles	Primary cluster	Tibet, Sichuan, Yunnan, Qinghai	7.08	2329.06	< 0.001	Primary cluster	Ningxia, Gansu, Qinghai, Shaanxi, Inner Mongolia	2.68	30.70	< 0.001
	Secondary cluster	Hunan	1.37	17.22	< 0.001	Secondary cluster	Yunnan	2.65	15.40	< 0.001
	Tertiary cluster	Zhejiang	1.35	12.52	< 0.001					

RIDs respiratory infectious diseases; PTB pulmonary tuberculosis; PLADs provincial-level administrative divisions; *RR* relative risk; *LLR*: log-likelihood ratio

**Table 3:** The APC and 95% *CI* for incidence of seven RIDs in the 31 PLADs during 2017–2021

Regions	Seven RIDs	Seasonal Influenza	PTB	Mumps	Scarlet Fever	Pertussis	Rubella	Measles
Chinese Mainland	−1.42 (−39.14 to 57.09)	0.72 (−54.07 to 130.48)	−7.53 (−12.31 to −2.79)*	−17.02 (−36.5 to 3.32)	−22.15 (−53.11 to 11.09)	−10.31 (−66.13 to 90.68)	29.35 (−74.18 to 540.89)	−40.87 (−61.18 to −24.32)*
Beijing	3.3 (−54.26 to 118.31)	8.73 (−59.48 to 181.79)	−2.56 (−8.2 to 3.12)	−15.65 (−28.01 to −4.36)	−32.95 (−72.99 to 20.15)	−16.43 (−61.29 to 46.19)	23.79 (−70.63 to 470.47)	−33.71 (−67.07 to 1.16)
Tianjin	−4.38 (−43.91 to 50.78)	11.2 (−58.64 to 171.06)	3.81 (−0.6 to 8.22)	−10.48 (−22.34 to 1.01)	−23.15 (−66.47 to 38.4)	−29.79 (−69.96 to 20.58)	28.32 (−63.41 to 364.83)	−27.06 (−65.17 to 22.54)
Hebei	−7.06 (−38.38 to 35.41)	−2.91 (−49.09 to 76.96)	−7.18 (−9.35 to −5.1)*	−18.49 (−39.59 to 2.33)	−23.92 (−65.95 to 35.56)	−21.49 (−65.47 to 41.66)	53.2 (−59 to 620.05)	−39.79 (−60.35 to −22.94)*
Shanxi	−7.9 (−20.13 to 5.41)	1.34 (−30.24 to 43.72)	−5.96 (−8.76 to −3.29)*	−22.69 (−37.77 to −8.63)	−18.45 (−41.08 to 5.26)	24.75 (−10.94 to 89.11)	5.49 (−53.76 to 91.8)	−35.51 (−69.2 to 1.38)
Inner Mongolia	−7.2 (−20.76 to 7.41)	2.6 (−40.99 to 77.18)	−7.46 (−19.74 to 5.22)	−6.79 (−16.79 to 3.26)	−26.12 (−47.55 to −8.05)	45.92 (1.45 to 156.7)	−24.09 (−81.82 to 201.26)	−10.22 (−22.74 to 2.37)
Liaoning	−8.08 (−19.94 to 3.79)	−0.18 (−43.22 to 69.66)	−5.07 (−12.33 to 2.22)	−3.94 (−16.84 to 9.28)	−31.94 (−70.88 to 18.11)	4.1 (−47.92 to 114.03)	−17.24 (−84.12 to 296.28)	−26.54 (−44.76 to −9.62)
Jilin	−12.01 (−24.4 to −0.21)	17.31 (−58.15 to 245.68)	−9.94 (−14.08 to −6.18)*	−2.92 (−21.57 to 17.26)	−35.49 (−70.92 to 4.93)	5.92 (−57.48 to 148.82)	133.02 (−49.12 to 2066.76)	−20.73 (−48.2 to 8.5)
Heilongjiang	−8.66 (−16 to −1.58)	30.64 (−38.16 to 220.52)	−9.01 (−12.55 to −5.77)*	2.68 (−27.83 to 42.18)	−30.94 (−71.79 to 24.39)	−7.21 (−64.86 to 109.94)	24.57 (−75 to 495.05)	−38.46 (−63.63 to −16.68)
Shanghai	−2.62 (−42.66 to 59.7)	5.49 (−57.21 to 172.51)	−3.85 (−6.8 to −1)	−14.1 (−33.15 to 5.33)	−30.53 (−69.14 to 19.13)	8.75 (−17.4 to 44)	1.91 (−88.07 to 658.95)	−21.81 (−67.81 to 51.69)
Jiangsu	2.22 (−43.05 to 87.84)	16.34 (−60.75 to 348.07)	−6.54 (−8.51 to −4.58)*	−3.01 (−36.94 to 47.53)	−11.95 (−58.27 to 61.54)	8.79 (−38.89 to 95.95)	−26.75 (−87.78 to 284.73)	−46.89 (−57.18 to −40.2)*
Zhejiang	3.97 (−60.39 to 199.76)	0.35 (−58.94 to 172.52)	−6.15 (−9.43 to −2.93)*	−14.94 (−23.23 to −6.76)	−19.32 (−40.7 to 2.42)	−10.04 (−69.29 to 104.63)	12.15 (−73.08 to 344.97)	−49.71 (−60.1 to −43.14)*
Anhui	−0.8 (−31.96 to 42.11)	9.14 (−56.49 to 194.4)	−7.77 (−9.41 to −6.29)*	−12.61 (−51.51 to 41.65)	−11.69 (−40.78 to 24.2)	23.95 (−38.29 to 161.19)	21.4 (−72.2 to 412.76)	−45.93 (−52.41 to −41.73)*
Fujian	−5.45 (−36.3 to 36.25)	−5.68 (−48.83 to 67.63)	−3.33 (−10.13 to 3.86)	−16.31 (−29.58 to −3.05)	−21.97 (−47.75 to 4.31)	16.9 (−49.49 to 200.23)	70.21 (−72.07 to 2360.79)	−16.91 (−63.72 to 60.15)
Jiangxi	−3.42 (−23.4 to 20.02)	7.18 (−46.14 to 122.46)	−6.9 (−11.43 to −2.64)*	−17.71 (−41.93 to 8.87)	1.95 (−21.6 to 31.05)	−2.36 (−45.75 to 70.38)	91.53 (−51.17 to 2508.55)	−14.72 (−42.49 to 20.89)
Shandong	−2.55 (−24.83 to 24.26)	13 (−21.21 to 71.32)	−7.22 (−9.13 to −5.42)*	−11.55 (−27.06 to 4.95)	−18.63 (−61.78 to 44.81)	−24.93 (−62.5 to 18.22)	23.16 (−70.25 to 395.26)	−13.58 (−45.31 to 26.96)
Henan	0.26 (−33.42 to 50.46)	0.01 (−56.95 to 154.43)	−10.31 (−15.06 to −5.84)*	−33.96 (−40.48 to −28.62)*	−25.87 (−58.91 to 13.23)	−6.77 (−62.52 to 102.6)	23.32 (−53.98 to 177.4)	−43.84 (−85.02 to 5.6)
Hunan	3.76 (−39.86 to 82.48)	−4.53 (−63.31 to 166.3)	−0.72 (−7.62 to 6.49)	−25.88 (−54.15 to 4.64)	−0.25 (−29.37 to 36.25)	−14.61 (−64.96 to 84.56)	54.36 (−74.74 to 1030.57)	−21.28 (−63.77 to 31.91)
Hubei	0.07 (−43.28 to 69.38)	3.52 (−58.9 to 164.21)	−6.43 (−8.59 to −4.43)*	−23.39 (−45.75 to −1.91)	−21.71 (−66.99 to 44.02)	−3.98 (−62.52 to 111.36)	10.52 (−75.63 to 407.3)	−36.98 (−81.46 to 26.26)
Guangdong	−7.59 (−49.67 to 61.53)	−3.24 (−60.88 to 123.68)	−11.81 (−14.86 to −8.99)*	−18.57 (−31.37 to −6.36)	−19.56 (−35.29 to −5.1)	−9.09 (−58.38 to 82.32)	40.78 (−75.71 to 956.34)	−27.36 (−48.98 to −7.2)
Guangxi	0.45 (−22.23 to 29.44)	7.57 (−33.4 to 82.94)	−5.91 (−9.76 to −2.26)*	−7.75 (−32.61 to 23.24)	−6.68 (−33.27 to 24.27)	−25.11 (−71.52 to 73.28)	−27.98 (−92.86 to 294.41)	−33.15 (−43.68 to −24.18)*
Hainan	−1.74 (−35.65 to 47.58)	−5.35 (−60.25 to 148.32)	−1.87 (−7.23 to 3.74)	−25.82 (−69.62 to 42.17)	−27.93 (−61.75 to 8.67)	−30.59 (−87.81 to 135.43)	7.06 (−74.84 to 278.84)	−27.8 (−87.76 to 169.37)
Chongqing	1.35 (−29.77 to 46.36)	−4.58 (−71.74 to 230.4)	−5.61 (−7.69 to −3.63)*	−20.30 (−26.81 to −14.73)*	−8.95 (−42.54 to 31.89)	−21.6 (−65.94 to 44.47)	−54.43 (−97.42 to 333.61)	−22.81 (−39.79 to −5.87)
Sichuan	9.58 (−32.2 to 84.54)	−10.47 (−50.7 to 102.82)	−2.98 (−5.56 to −0.44)	−13.03 (−34.16 to 9.97)	−8.15 (−27.14 to 12.34)	15.14 (−47.77 to 149.84)	23.55 (−76.77 to 577.48)	−66.18 (−81.62 to −56)*
Guizhou	−9.03 (−14.63 to −3.35)	5.26 (−31.76 to 65.73)	−8.4 (−11.94 to −4.97)*	−25.39 (−37.62 to −15.39)*	−7.13 (−35.68 to 28.78)	−7.01 (−64.55 to 114.35)	18.68 (−76.86 to 341.21)	−56.62 (−69.49 to −49.03)*
Yunnan	3.89 (−23.91 to 41.43)	9.27 (−45.03 to 138.87)	−0.89 (−4.77 to 3.01)	−1.7 (−40.24 to 56.84)	−1.99 (−22.36 to 21.67)	79.74 (46.24 to 163.19)*	−9.64 (−69.62 to 166.09)	−24.4 (−59.32 to 15.55)
Tibet	−2.72 (−11.86 to 6.81)	57.99 (−13.77 to 387.68)	−4.6 (−13.48 to 4.7)	−4.25 (−53.37 to 80.76)	−19.19 (−34.63 to −4.51)	−2.09 (−58.27 to 129.38)	8.56 (−16.61 to 42.89)	−82.74 (−94.65 to −77.08)*
Shaanxi	−0.61 (−52.76 to 110.89)	−16.34 (−75.7 to 190.95)	−8.64 (−14.29 to −3.31)*	−17.30 (−33.29 to −1.63)*	−18.07 (−48.73 to 16.45)	−16.82 (−80.73 to 132.2)	98.16 (−77.99 to 3298.54)	−25.77 (−58.57 to 14.05)
Gansu	−7.19 (−27.15 to 16.12)	−0.42 (−25.76 to 31.95)	−14.76 (−24.25 to −6.06)*	−1.05 (−54.68 to 103.67)	−20.18 (−51.34 to 13.11)	−7.6 (−62.88 to 97.44)	−35.98 (−93.68 to 460.92)	−40.52 (−64.09 to −21.87)*
Qinghai	−7.71 (−21.47 to 7.29)	−3.25 (−37.93 to 46.07)	−10.56 (−20.95 to −0.22)*	−4.55 (−48.83 to 68.07)	15.92 (−9.33 to 51.56)	12.16 (−35.45 to 99.28)	74.74 (−10.65 to 489.68)	−59.3 (−79.09 to −46.41)*
Ningxia	−12.5 (−25.72 to 1.87)	−8.92 (−39.07 to 32.69)	−12 (−16.19 to −8.15)*	−7.89 (−12.17 to −3.79)*	−21.18 (−53.21 to 15.28)	21.8 (−23 to 137.02)	53.96 (−61.89 to 647.9)	−45.72 (−70.17 to −25.02)*
Xinjiang	−21.42 (−52.16 to 15.59)	−2.86 (−64.5 to 152.5)	−23.51 (−40.7 to −6.25)	−25.76 (−39.74 to −12.67)	−32.99 (−70.65 to 14.31)	2.1 (−72.51 to 226.96)	1.54 (−63.34 to 150.71)	−52.66 (−64.04 to −45.61)*

APC annual percentage changes; *CI* confidence interval; RIDs respiratory infectious diseases; PTB pulmonary tuberculosis; PLADs provincial-level administrative divisions. Notes: The unit of APC is %. * represent *P* value < 0.05

Spatial distributions for individual RIDs were also diverse among PLADs. Seasonal influenza showed four clusters covering four PLADs in 2017 and four clusters covering 13 PLADs in 2021, with the most likely cluster located in Guangdong (*RR* = 3.68, *P* < 0.001) in 2017 and spanning 10 PLADs (Guizhou, Chongqing, Yunnan, Guangxi, Sichuan, Hunan, Guangdong, Hainan, Hubei and Shaanxi; *RR* = 2.42, *P* < 0.001) in 2021 (Table 2). PTB exhibited three clusters covering 14 PLADs in 2017, primarily within Xinjiang, Qinghai and Tibet (*RR* = 3.18, *P* < 0.001). In 2021, four clusters spread across 15 PLADs, with the most likely cluster encompassing 10 PLADs (Guizhou, Chongqing, Yunnan, Guangxi, Sichuan, Hunan, Guangdong, Hainan, Hubei and Shaanxi; *RR* = 1.66, *P* < 0.001) (Table 2). PTB incidence displayed a decreasing trend in 19 PLADs, with the highest APC in Gansu (APC = −14.76%, *P* = 0.009), mirroring the national pattern (Table 3). Mumps presented with the most likely cluster spanning 16 PLADs (*RR* = 2.24, *P* < 0.001) in 2017, while two clusters covered 14 PLADs in 2021 with the most likely cluster encompassing 13 PLADs (*RR* = 1.97, *P* < 0.001) (Table 2). National trends largely showed no significant change of mumps, except for notable decreases in Henan (APC = −33.96%, *P* = 0.004), Guizhou (APC = −25.39%, *P* = 0.02), Chongqing (APC = −20.30%, *P* = 0.008), Shaanxi (APC = −17.30%, *P* = 0.04), and Ningxia (APC = −7.89%, *P* = 0.03) (Table 3). Scarlet fever demonstrated four clusters across 15 PLADs in 2017 and five clusters covering 16 PLADs in 2021. The most likely cluster included nine PLADs (Heilongjiang, Jilin, Liaoning, Beijing, Tianjin, Shandong, Hebei, Inner Mongolia and Shanxi; *RR* = 2.52, *P* < 0.001) in 2017 and six PLADs (Ningxia, Gansu, Qinghai, Shaanxi, Inner Mongolia and Shanxi; *RR* = 2.13, *P* < 0.001) in 2021 (Table 2). Scarlet fever incidence displayed no significant trend in the 31 PLADs, aligning with the national pattern (Table 3). Pertussis exhibited three clusters across four PLADs in 2017, with the most likely cluster encompassing Shandong and Tianjin (*RR* = 8.04, *P* < 0.001). In 2021, two clusters spanned seven PLADs, with the most likely cluster covering six PLADs (Chongqing, Sichuan, Guizhou, Shaanxi, Yunnan and Hunan; *RR* = 3.66, *P* < 0.001) (Table 2). Pertussis incidence displayed no significant trend in most PLADs, except for an increase in Yunnan (APC = 79.74%, *P* = 0.02) (Table 3). Rubella presented with three clusters spanning seven PLADs in 2017, with the most likely cluster in Fujian (*RR* = 4.53, *P* < 0.001). In 2021, four clusters covered six PLADs, with the most likely cluster in Qinghai (*RR* = 42.06, *P* < 0.001) (Table 2). Rubella incidence rate showed no significant trend across PLADs (Table 3). Measles exhibited three clusters across six PLADs in 2017 and two clusters spanning six PLADs in 2021. The most likely cluster included four PLADs (Tibet, Sichuan, Yunnan and Qinghai; *RR* = 7.08, *P* < 0.001) in 2017 and five PLADs (Ningxia, Gansu, Qinghai, Shaanxi and Inner Mongolia; *RR* = 2.68, *P* < 0.001) in 2021 (Table 2). Measles incidence displayed decreasing trends in 12 PLADs, with Tibet showing the greatest decrease (APC = −82.74%, *P* < 0.001), consistent with the national trend (Table 3).

### Characteristics of laboratory-confirmed and clinically diagnosed cases

The five-year average incidence rate of laboratory-confirmed cases for all seven RIDs combined was 35.64/100,000, peaking at 96.70/100,000 in 2019 (Fig. 1A). Among the individual RIDs, the incidence rates of laboratory-confirmed cases for scarlet fever (APC = −24.19%, 95% *CI*: −45.40 to −5.15%, *P* = 0.02) and measles (APC = −38.78%, 95% *CI*: −58.00 to −22.20%, *P* = 0.03) decreased, while the remaining RIDs showed no significant trends from 2017 to 2021 (Table 1 and Fig. 5).

**Fig. 7:**
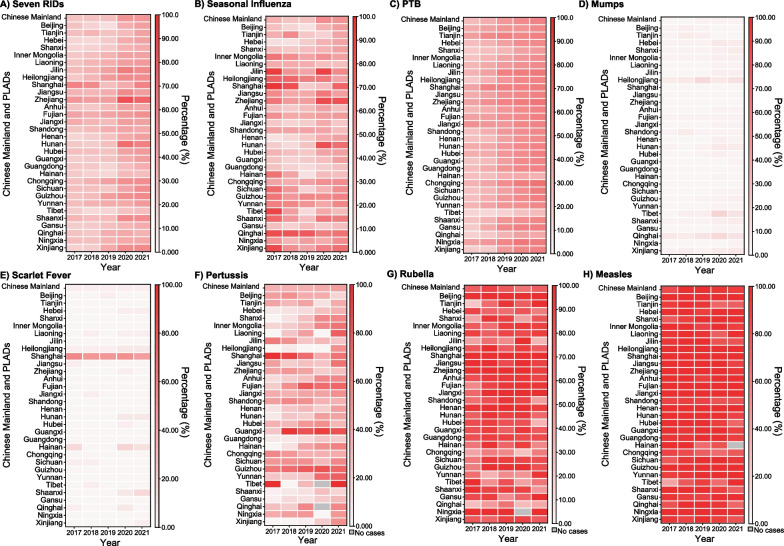
The percentage of laboratory-confirmed cases for seven RIDs during 2017–2021. *PLADs*: Provincial-level administrative divisions; *RIDs*: Respiratory infectious diseases; *PTB*: Pulmonary tuberculosis

The proportion of laboratory-confirmed cases among all seven RIDs increased from 26.33% in 2017 to 48.21% in 2021, with a peak of 48.96% in 2020 (Fig. 7A). Notably, different RIDs exhibited varying proportions of laboratory-confirmed cases, with measles and rubella having higher proportions, while mumps and scarlet fever displayed lower proportions (Fig. 7D, E, G, H). In 2021, the national proportion of laboratory-confirmed cases for seasonal influenza reached 49.61%, with significant variations among PLADs, ranging from 82.63% in Zhejiang to 24.54% in Jiangxi (Fig. 7B). For PTB, the proportion of laboratory-confirmed cases varied across PLADs, with the highest in Chongqing (65.09%) and the lowest in Tibet (30.56%) in 2021 (Fig. 7C). Mumps displayed relatively low proportions of laboratory-confirmed cases across PLADs, with higher values in Heilongjiang (10.40%), Tibet (9.26%), Qinghai (8.35%) and Xinjiang (7.13%) in 2021 (Fig. 7D). Scarlet fever showed a similar pattern, with Shanghai having the highest proportion of laboratory-confirmed cases (Fig. 7E). Pertussis exhibited a nationwide proportion of 44.94% in 2021, with varying proportions among PLADs due to differing case reporting (Fig. 7F). Rubella and measles both had a high proportion of laboratory-confirmed cases, with national rates of 75.12% and 91.11% respectively in 2021 (Fig. 7G, H).

## Discussion

This study primarily aimed to uncover the demographic, temporal, seasonal, and spatial distribution patterns of seven RIDs using recent national surveillance data. Additionally, we conducted a more comprehensive analysis of RIDs diagnosis categories, gender distribution, and occupation distribution, aspects that were not covered in our previous study [9]. The results indicate a significant decrease in the incidence of PTB and measles, while other RIDs showed no significant temporal trends, both for laboratory-confirmed and clinically diagnosed cases. Variations in distribution were observed across different populations for different RIDs, with PTB predominantly affecting the elderly and children and students being more susceptible to other RIDs. Furthermore, the spatial–temporal distribution and clustering characteristics varied for different RIDs across different regions during 2017–2021. Our findings hold valuable insights for enhancing RIDs control and prevention efforts. Nevertheless, the multifaceted factors influencing these epidemiological characteristics warrant further in-depth exploration.

This study underscores that the incidences of seasonal influenza, PTB, mumps, and scarlet fever remain substantial, while pertussis, rubella, and measles have reached lower levels. Findings from our study align with previous research on various aspects of RIDs characteristics in different areas and time periods [13–16, 20]. Through joinpoint regression analysis, we have established that the overall incidence rate of the seven RIDs remained relatively stable over the five-year period. Incidence peaks were observed in 2019 for seasonal influenza, mumps, scarlet fever, pertussis, and rubella, with decreasing trends observed for PTB and measles from 2017 to 2021. Similar patterns have been noted in other studies addressing specific aspects of RIDs [9, 11, 12, 16, 20–22].

A staggering 12 million reported cases of seven RIDs were documented in the mainland of China between 2017 and 2021. Among these, the incidences of all seven RIDs were notably higher in males than females, particularly for PTB, mumps, and scarlet fever, findings consistent with several reports [15, 20, 23]. Our study reveals that the incidence rate of the total seven RIDs predominantly occurred in the population under 15 years old, with seasonal influenza, mumps, scarlet fever, and pertussis being particularly prevalent. A parallel study on seasonal influenza identified students as high-risk groups, and schools as susceptible sites for cluster epidemics [24]. Furthermore, preschool children remained at high risk for seasonal influenza, pertussis, and measles, warranting continuous surveillance efforts [15, 24]. Interestingly, our study also uncovers higher rubella incidence rates among students and those aged 15–39 years old, a phenomenon that diverges from earlier findings [13]. A report on rubella during 2018–2019 concurs with our study's findings, suggesting that this shift in high-incidence age groups could be attributed to vaccine-induced immunity [25]. Consistent with other research, adults bore the highest burden of PTB, with individuals aged 65 and above exhibiting the highest incidence rates [9, 23]. Our results also indicate that farmers faced a higher risk of PTB, a trend potentially influenced by limited health services and patient management in rural areas [23], compounded by a lack of disease prevention awareness contributing to PTB transmission [26].

Significantly, our study highlights a decrease in the incidences of PTB and measles across the Chinese mainland, a trend supported by previous reports [11, 16, 20, 27]. The progressive decline in PTB incidence can be attributed to multifaceted efforts [28, 29], with a noticeable reduction in the disease burden observed in most PLADs, attesting to successful PTB control and prevention measures. This positive outcome is further affirmed by the increasing proportion of laboratory-confirmed cases, indicative of improved PTB control and prevention capabilities. However, we did observe persistent high PTB incidence rates in certain regions, including Tibet, Xinjiang, Hainan, and Hunan, a trend corroborated by other studies to some extent [6, 13]. This highlights the pressing need for continued financial investment and capacity-building endeavors to effectively combat PTB, particularly in areas where the disease remains prevalent. Similarly, measles incidence remained at low levels, reflecting the substantial progress made through comprehensive preventive strategies, such as China’s Measles Elimination Action Plan [11], bolstered by heightened surveillance sensitivity and other disease prevention and control measures [15, 30].

The evolving healthcare landscape, marked by enhanced diagnostic capabilities and surveillance systems, particularly in diagnostic proficiency, has contributed to improved laboratory diagnostics in the mainland of China [15, 29, 30]. The proportion of laboratory-confirmed cases increased from 26.33% in 2017 to 48.21% in 2021. Notably, cases of measles and rubella were overwhelmingly laboratory-confirmed, while the majority of other RIDs cases were clinically diagnosed, a pattern that holds true for mumps and scarlet fever. Clinically reported cases can enhance reporting sensitivity and surveillance, with subsequent laboratory confirmation serving to enhance reporting accuracy and overall data quality. The development of tailored diagnostic and monitoring tools, aligned with the specific characteristics of individual RIDs and laboratory testing capabilities, can significantly enhance the sensitivity and accuracy of RIDs surveillance.

In summary, infectious diseases, particularly RIDs, remain critical public health concerns. Effective control and prevention strategies are of paramount importance. Leveraging recent national surveillance data from the NNDRS, spanning 31 PLADs and featuring detailed information, this study has provided insights into the epidemiological characteristics of clinically diagnosed and laboratory-confirmed RIDs cases. Notably, while PTB and measles incidence rates have significantly declined, other RIDs have exhibited stable or fluctuating trends over the 2017–2021 period. These findings offer crucial guidance for shaping tailored strategies for RIDs control and prevention. However, the five-year duration of available surveillance data and potential variations in reporting across regions and levels present certain limitations.

## Conclusions

The incidence rates of seasonal influenza, PTB, mumps, and scarlet fever remain high, whereas pertussis, rubella, and measles have reached lower levels. The reported incidence rates of PTB and measles have experienced significant declines between 2017 and 2021. Peaks in reported incidence rates for seasonal influenza, mumps, scarlet fever, pertussis, and rubella were observed in 2019. Each of the seven RIDs demonstrates distinct epidemiological characteristics. While significant progress has been made in RIDs control and prevention, challenges persist. Urgent measures are needed to strengthen surveillance efforts and develop effective digital/intelligent systems for precise RIDs surveillance, early detection of emerging or re-emerging events, and prompt responses.

### Supplementary Information

**Additional file 1: Figure S1.** The spatial distributions for incidence rates of the seven RIDs in 2017 and 2021.

## Data Availability

The data is from the National Notifiable Disease Reporting System (NNDRS), and the application is based on the requirement of NNDRS.

## Abbreviations

RIDs: Respiratory infectious diseases
PTB: Pulmonary tuberculosis
NNDRS: National notifiable disease reporting system
APC: Annual percentage changes
PLADs: Provincial-level administrative divisions
GSM: Grid search method
BIC: Bayesian information criterion
*RR*: Relative risk
*LLR*: Log-likelihood ratio
