# Supplementary figures and images for: Epidemiological characteristics of seven notifiable respiratory infectious diseases in the mainland of China: an analysis of national surveillance data from 2017 to 2021
Source: Infect Dis Poverty. 2023 Nov 13;12:99. doi: 10.1186/s40249-023-01147-3 (PMC10642048; doi:10.1186/s40249-023-01147-3)

A) Seven RIDs

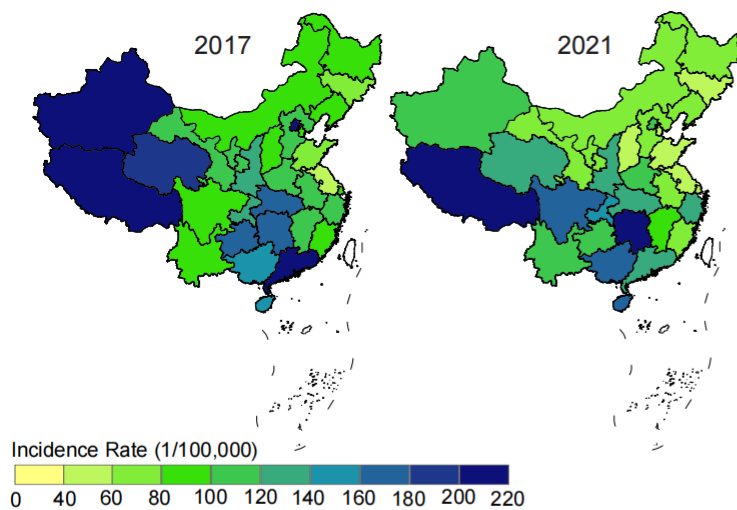

B) Seasonal Influenza

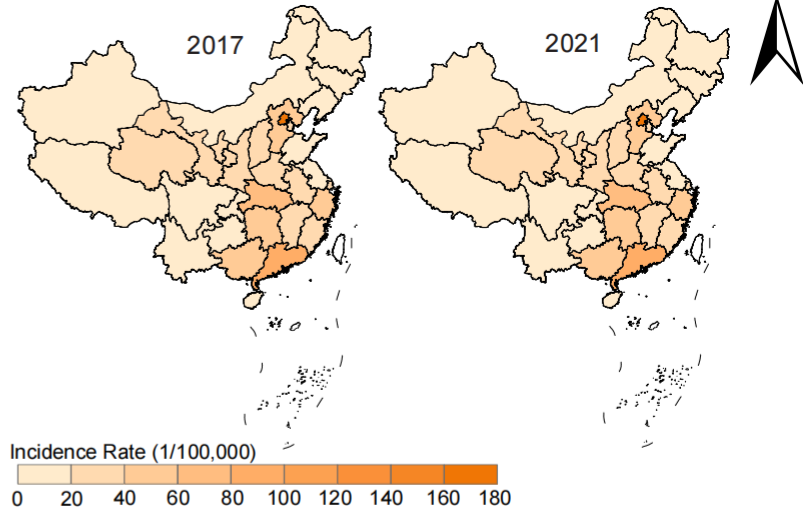

C) PTB

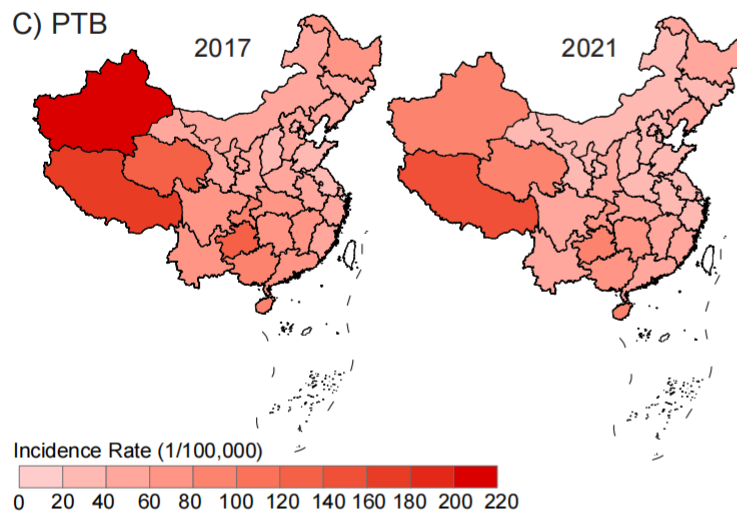

D) Mumps

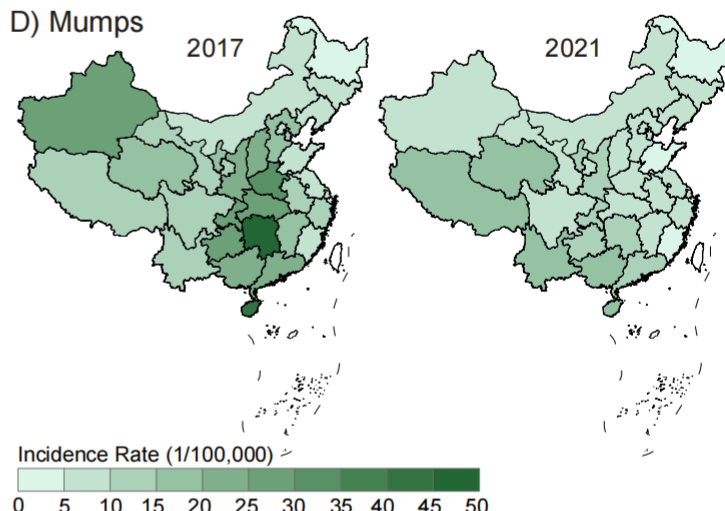

E) Scarlet Fever

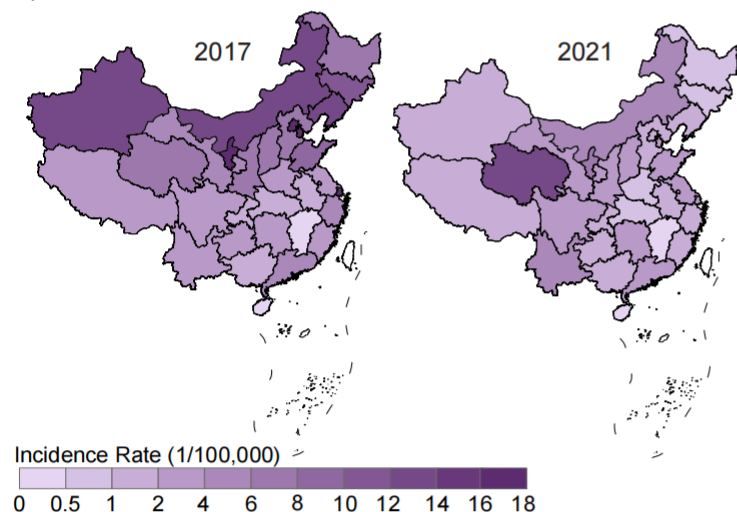

F) Pertussis

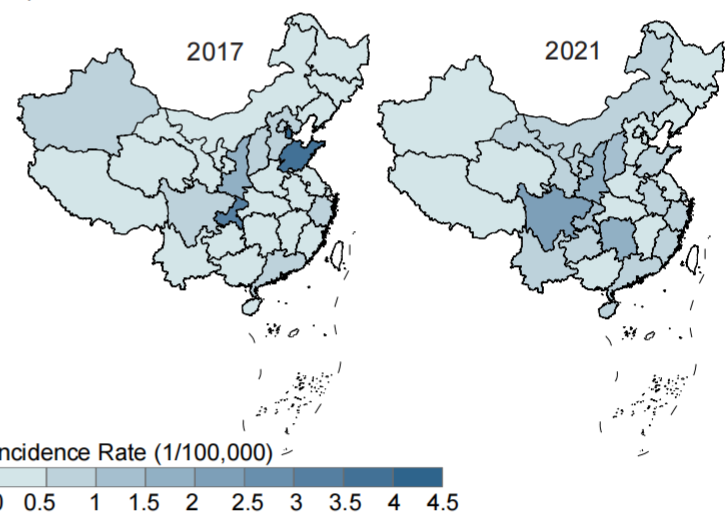

G) Rubella

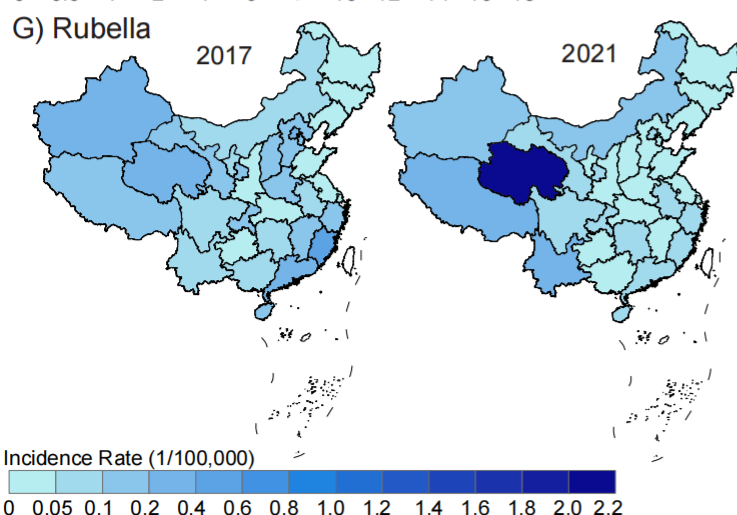

H) Measles

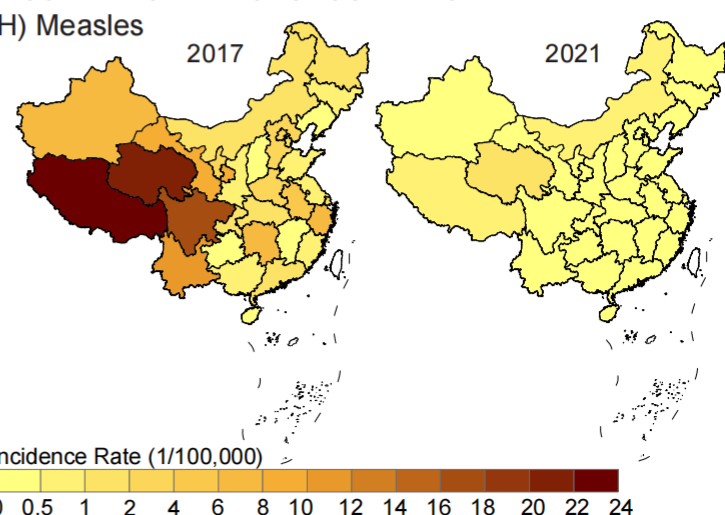

Supplement: Supplementary file 1 — Additional file 1: Figure S1. The spatial distributions for incidence rates of the seven RIDs in 2017 and 2021. [file 40249_2023_1147_MOESM1_ESM.pdf]
